# Oral Dosing of Chemical Indicators for *In Vivo* Monitoring of Ca^2+^ Dynamics in Insect Muscle

**DOI:** 10.1371/journal.pone.0116655

**Published:** 2015-01-15

**Authors:** Ferdinandus, Satoshi Arai, Shin’ichi Ishiwata, Madoka Suzuki, Hirotaka Sato

**Affiliations:** 1 School of Mechanical and Aerospace Engineering, Nanyang Technological University, Singapore, Singapore; 2 WASEDA Bioscience Research Institute in Singapore (WABIOS), Singapore, Singapore; 3 Organization for University Research Initiatives, Waseda University, Tokyo, Japan; 4 Department of Physics, Faculty of Science and Engineering, Waseda University, Tokyo, Japan; University of Tours, FRANCE

## Abstract

This paper proposes a remarkably facile staining protocol to visually investigate dynamic physiological events in insect tissues. We attempted to monitor Ca^2+^ dynamics during contraction of electrically stimulated living muscle. Advances in circuit miniaturization and insect neuromuscular physiology have enabled the hybridization of living insects and man-made electronic components, such as microcomputers, the result of which has been often referred as a Living Machine, Biohybrid, or Cyborg Insect. In order for Cyborg Insects to be of practical use, electrical stimulation parameters need to be optimized to induce desired muscle response (motor action) and minimize the damage in the muscle due to the electrical stimuli. Staining tissues and organs as well as measuring the dynamics of chemicals of interest in muscle should be conducted to quantitatively and systematically evaluate the effect of various stimulation parameters on the muscle response. However, existing staining processes require invasive surgery and/or arduous procedures using genetically encoded sensors. In this study, we developed a non-invasive and remarkably facile method for staining, in which chemical indicators can be orally administered (oral dosing). A chemical Ca^2+^ indicator was orally introduced into an insect of interest via food containing the chemical indicator and the indicator diffused from the insect digestion system to the target muscle tissue. We found that there was a positive relationship between the fluorescence intensity of the indicator and the frequency of electrical stimulation which indicates the orally dosed indicator successfully monitored Ca^2+^ dynamics in the muscle tissue. This oral dosing method has a potential to globally stain tissues including neurons, and investigating various physiological events in insects.

## Introduction

The fusion of microcomputers and living insects, resulting in Cyborg Insects, also known as Insect–Machine Hybrid Systems or Living Machines [1], which include biological actuators and biological machines, has been a popular research topic owing to the production of miniature digital circuits and study of insect neuromuscular physiology [2, 3]. Ultra-small, radio-enabled, neuromuscular recorders and stimulators have been designed and manufactured to measure EMG (electromyogram) and to induce certain motor actions even in untethered and free flight situations [3–17]. Most attempts toward Cyborg Insects were to find which muscular or neural sites should be stimulated to induce a specific motor action and by what electrical signals (amplitude, frequency, and waveform). A challenge that remains is to regulate the induced motion to draw a user-determined path in a feedback controlled manner, where motor actions are appropriately graded by altering certain stimulation parameters [14]. To develop a model based feedback control system, we need to quantitatively investigate the effect of a given parameter on motor actions. To this end, it is desirable to visualize the muscle contractions, in other words, to monitor the dynamics of the intracellular Ca^2+^ levels (a significant second messenger molecule for muscle contraction) *in vivo* during electrical stimulation.

*In vivo* bioimaging is a promising way to gain insights into the physiological effects of exogenously applied electrical stimuli on living muscles. Advances in microscopic technology and the development of fluorescence labeling technology enable us to observe the dynamics of signaling molecules and intracellular components at cellular and tissue levels [18]. In particular, recent advances in the development of fluorescence imaging techniques provide various tools to visualize the dynamics of Ca^2+^, an ion widely considered to be a significant second messenger molecule.

Ca^2+^ dynamics in living muscle cells are key factors for understanding muscular physiology. The sarcoplasmic reticulum (SR) releases Ca^2+^ to bind with troponin and alter the configuration of tropomyosin, which then allows the formation of cross-bridges between actin and myosin filaments to enable muscle contraction [19, 20]. Gordon and Dickinson reported the active role of Ca^2+^ in controlling the mechanical power output from flight muscles in *Drosophila melanogaster* [21]. Since Ca^2+^ is a major regulator for the motor action of muscle cells, we were motivated to visualize and further characterize Ca^2+^ dynamics in muscles in order to develop stimulation protocols that would be able to reliably induce the desired motor actions, all with the aim of producing an advanced level Cyborg Insect. Ca^2+^ imaging is a key technology not only for the study of muscular physiology in developing Cyborg Insects but also for a comprehensive physiological observation of the sensory units and the brain. Ca^2+^ imaging is popularly used in insect optophysiological studies to visualize neuronal activity in the antennal lobes of various insects. For instance, Joerges et al. [22] and Galizia and Kimmerle [23] utilized a confocal microscope to visualize Ca^2+^ dynamics representing the neuronal responses to a variety of odors in the glomerulus of the antennal lobes of honeybees. Wang et al. [24] employed a two-photon Ca^2+^ imaging technique to map out the glomerular activation evoked by different odors in the antennal lobe of flies. Overall, there are many great needs for Ca^2+^ imaging techniques in various insect neuromuscular physiology studies.

Two types of Ca^2+^ indicators (i.e. genetically encoded and chemical indicators) have been investigated, with each indicator presenting both advantages and disadvantages [25–27]. Genetically encoded indicators, such as Cameleon and GCaMP, use specifically designed vectors that contain special sequences that genetically target organelles and tissues of interest. Although some biological laboratories employed these methods using transgenic animals [28, 29], the application of these methods are still limited to a few animal species, such as: mouse, fly, pig, and sheep, owing to the genetic complexity of the organism of interest [30]. A wide variety of chemicals to monitor Ca^2+^ concentrations without the needs for genetic modification have been synthesized to date. There are commercially available Ca^2+^ indicators with a wide range of Ca^2+^ affinities including Calcium Green-1, Fluo-3, Fluo-4, and Fluo-8. However staining living tissues and cells with chemical indicators presents its own challenges, especially in *in vivo* applications. Regularly employed protocols for *in vivo* staining with chemical indicators need to immobilize the animal of interest and to excise and expose the target tissues in order to inject, drip or spray the chemicals. The target cells and tissues could be crucially damaged during such a staining process, especially for small animals like insects, which would consequently cause a significant physiological difference between the stained and intact animals.

Herein, we propose a non-invasive and facile method to load chemical indicators into the tissues of living insects via jelly (insect food) containing the chemical indicators of interest, which is subsequently fed to living insects. The chemical indicators are not injected into dissected tissues, but an intact insect is simply fed with the jelly to orally introduce the chemical indicators from the mouth to the fore-, mid-, and hindguts via the esophagus. In general, insects digest food in the midgut, in which macromolecules are broken down into smaller components for absorption in the mid- and hindguts. Unlike in vertebrates, once molecules are absorbed, they are circulated around the insect body through the flow of hemolymph (insect body fluid), which are then delivered into various insect tissues [19]. Similarly, chemical indicators for physiological investigations and experiments, such as the Ca^2+^ indicator, could be orally dosed and delivered to muscle tissues of living insects. In the present study, we developed and tested the oral dosing method by using a variety of chemical dyes and indicators. Unlike the more commonly used methods to load chemical indicators, the oral dosing method does not require anaesthetizing and immobilizing the animal, which can freely and naturally behave and feed during the dosing, loading, and staining periods.

After we examined a variety of chemical indicators for staining muscles via the oral dosing method, we chose a chemical Ca^2+^ indicator (Fluo-8) and a cell-staining chemical (Cell Tracker) to investigate the effect of exogenously applied electrical stimuli on the stained muscle. We then conducted *in vivo* monitoring of Ca^2+^ dynamics in insect muscles in response to electrical stimulus.

## Materials and Methods

### Study Animal

We used *Mecynorrhina torquata* (Coleoptera: Scarabaeidae) as our insect model. *Mecynorrhina torquata* beetles are approximately 60 mm long and 8 g weigh. The beetles were kept in separate plastic terrariums (20 cm × 15 cm × 15 cm) with tissue paper at the bottom and were fed with a cup of sugar jelly (Lai Bao Food Co., Ltd) every 2–3 days. The temperature and relative humidity in the terrariums were maintained at 23°C and 50%, respectively [10]. The use of beetles is permitted by the Agri-Food & Veterinary Authority of Singapore (AVA, HS code: 01069000, Product code: ALV002). Invertebrates including insects are exempt from the ethics for animal experimentation according to National Advisory Committee for Laboratory Animal Research (NACLAR) Guidelines.

### Chemicals

Fluo-8, AM was acquired from AAT Bioquest, Inc. Cell Tracker Orange CMRA; Rhodamine 123; bis-(1,3-dibutylbarbituric acid) trimethine oxonol [DiBAC_4_(3)] and Grace’s insect medium (1X), unsupplemented, were obtained from Life Technologies. Rhodamine B was purchased from Sigma-Aldrich. Dimethyl sulfoxide (DMSO) was acquired from Kanto Chemical Co. Inc. Fluo-8, AM (50 µg), Cell Tracker Orange CMRA (50 µg), Rhodamine 123 (3 mg), and DiBAC_4_(3) (5 mg) were each diluted with DMSO to produce 1 mM stock solutions. Rhodamine B (5 mg) was diluted in water to produce 1 mM stock solution.

### Electrical Stimulation Apparatus and Fluorescence Microscopy

**Figure 1:**
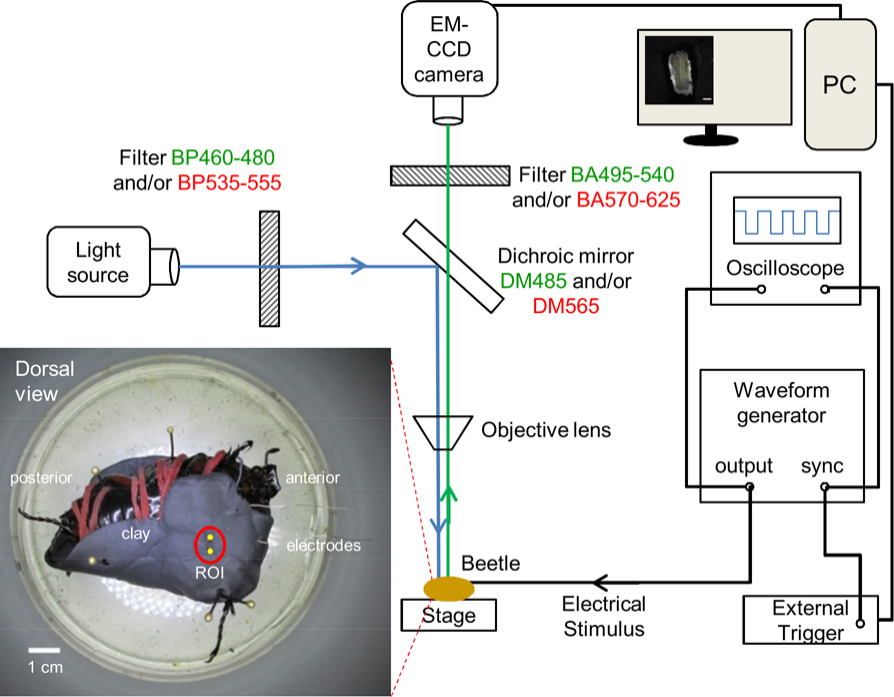
Illustration of fluorescence microscopy and electrical stimulation setups. The filter and mirror set in green font is used to observe Fluo-8, Rhodamine 123, and DiBAC_4_(3). The filter and mirror set in red font is used to observe Cell Tracker and Rhodamine B. *Inset*, dorsal view of the beetle fixed upside down on dissection plate using clay, rubber bands (in red), and insect pins. Two PFA-coated thin silver wire electrodes (one working electrode and one counter electrode) were inserted into the ends of the muscle that are marked by 2 yellow dots. The insertion depth is approximately 2 mm, as measured from the outer cuticle. The red circle indicates the observation window that was used to observe the flexion muscle, which located inside the femur of the beetle leg.

An Agilent 33220A 20-MHz function/arbitrary waveform generator was used to perform electrical stimulation. An Agilent DSO 1004A 60 MHz oscilloscope was used to monitor the waveform that was generated during stimulation. An Olympus MVX10 Macro Zoom System Microscope with an objective lens (MVPLAPO 1X, N.A. = 0.25) was used for observation. A BP460–480 excitation filter, a DM485 dichroic mirror, and a BA495–540 barrier filter were used to observe Fluo-8, Rhodamine 123, and DiBAC_4_(3). A BP535–555HQ excitation filter, a DM565HQ dichroic mirror, and a BA570–625HQ barrier filter were used to observe Cell Tracker Orange CMRA and Rhodamine B. An Olympus 130 W U-HGLGPS light guide-coupled illumination system was used as the fluorescent light source. The optical filters, mirrors, and fluorescent light source were purchased from Olympus. An electron-multiplying charge-coupled device (EM-CCD) camera (iXon3 897; Andor Technology) and Andor iQ2.7 software (Andor Technology) were used to capture images. An external trigger device (ER-BOBD-100; Andor Technology) was used to record the electric stimulation that was triggered by the waveform generator. The size of the observation field was 4.1×4.1 mm in 512×512 pixels. Two-dimensional images were obtained with exposure time of 30 ms and time series images were acquired with a rate of 45–47 ms per frame, or approximately 22 Hz. Fig. 1 illustrates the experimental setup that was utilized in this study.

### Oral Dosing of Chemical Indicators and Electrode Implantation

The beetles were fed with home-made jelly that contained the chemical indicators. The jelly was made from mixing 153.5 mg of extra fine caster sugar (SIS sugar) in 500 mL of deionized water and was heated until all of the sugar dissolved. Three grams of agar-agar (Jim Willie Trading Co.) was added to the sugar solution while the heat was maintained, and the mixture was then brought to a boil. Afterward, the heat was turned off, and the solution was cooled to room temperature. The jelly solution was then transferred to small containers and placed in the freezer to solidify. Once the jelly solidified, it was mechanically mashed into small pieces using a spoon prior to mixing with the chemical indicators. Stock solutions of chemical indicators were then mixed with the mashed jelly (1 mL) to produce a final concentration of 60 µM of indicator in the mixture. The beetles were then subsequently fed with the jelly mixture (60 µM, 1 mL) for 2 days. One chemical indicator is mixed in one jelly solution, except for Fluo-8 and Cell Tracker which were mixed together. After the beetle was fed with the mixed jelly for 2 days, it was fixed on a dissection plate using rubber bands, insect pins, and clay while leaving the leg of interest free (a micrograph in Fig. 1). A small observation area was made on the side of the femur to observe the flexion muscle. One microliter of Grace’s insect medium was dropped to the opening to prevent drying of the observation area. The electrode used was a paraformaldehyde-coated thin silver wire that was acquired from A-M systems. The inserted portion of the silver wire was flamed to remove the insulation. The electrically stimulated sites were either on the right side of the muscle (S1B Fig.) or on the left side.

### Data Acquisition, Electrical Stimulation, and Data Analysis

Once the beetle was set on the stage under the microscope, data acquisition was started by using the filter setting for the indicator of choice. To investigate the effect of electrical stimulus on Ca^2+^ concentration in muscle, the beetles which were fed with Fluo-8 and Cell Tracker-mixed jelly were further examined under electrical stimulation, and data was acquired as a set of time series images using Fluo-8 filter setting followed by another set of images using Cell Tracker filter setting with 5–10 minutes time interval between each acquisition. Electrical stimulation began with multiple-pulse trains (50 Hz, 10% duty cycle, 2 V) for 3 seconds at 10 second timestamp, and then followed with varying stimulation frequencies to complete one stimulation cycle. A stimulation cycle is defined as a set of varying frequencies of multiple-pulse trains (50 Hz, 10 Hz, 1 Hz, 100 Hz, 1 Hz and 50 Hz with constant 10% duty cycle, 2 V) applied for 3 seconds for each frequency and 27 seconds resting time (no stimulation) in between frequencies. One complete data acquisition of one stimulation cycle took about 3 minutes.

ImageJ software (National Institutes of Health) was used to analyze the images after capture. The fluorescence intensity of each dosed beetle leg was calculated from the average of the intensity measured from the two regions of interest (ROIs, S1A Fig.). These ROIs were chosen such that the center part of the leg, which is not part of the muscle, and the cuticles on the periphery, which give significantly higher fluorescence signal compared to the muscle, were excluded for analysis. For the purpose of measuring the fluorescence intensity change during electrical stimulation on beetle leg, the intensity was only measured at the ROI adjacent to the stimulated site (S1B Fig.).

## Results and Discussion

### Staining of Living Insect Leg Muscle with Orally Dosed Chemical Indicators

First, we tested dosing beetles with different chemical indicators to investigate the effectiveness of the novel oral-dosing method for in vivo staining. Insect has unique digestion and circulation systems in which the absorbed molecules from the guts are circulated around the body and directly can be distributed into tissues [19]. Thus our hypothesis is that these chemical indicators could be able to stain insect leg muscle by oral administration via food. The survivorship of the target animal, *M. torquata*, after the oral dosing of the tested chemical indicators was sufficient for our purposes. All of the beetles that were fed with either Rhodamine B, Rhodamine 123, or DiBAC_4_(3) [N = 5 beetles for each indicator] and 9 out of 10 beetles that were fed with the Fluo-8 and Cell Tracker mixed jelly were still active for at least 1 week from the start of oral dosing.

**Figure 2:**
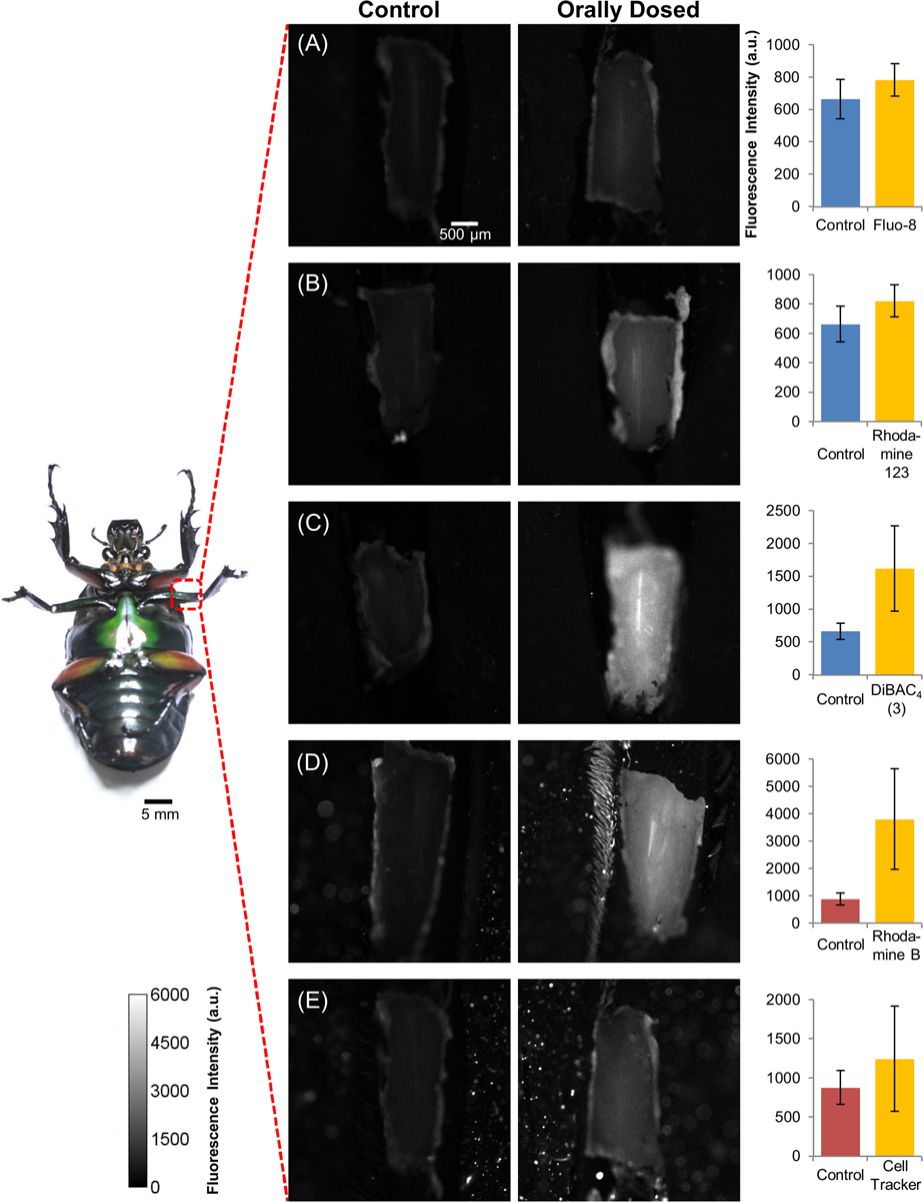
Difference in fluorescence intensity of insect flexion leg muscles of the control beetle and the beetle after oral dosing with chemical indicators. (A) Fluo-8; (B) Rhodamine 123; (C) DiBAC_4_(3); (D) Rhodamine B; and (E) Cell Tracker. The Fluo-8, Rhodamine 123, and DiBAC_4_(3) dosed beetle leg was observed under 460–480 nm excitation light and fluorescence emitted was collected within 495–540 nm. The Rhodamine B and Cell Tracker dosed beetle leg was observed under 535–555 nm excitation light and fluorescence emitted was collected within 570–625 nm. Fluorescence intensity was measured at the 2 regions of interest (ROIs) shown in S1A Fig. The images obtained were digitized by ImageJ software, and the averaged intensity is shown in each bar graph. The graphs in the right column show the fluorescence intensities of each beetle leg dosed with different chemical indicators (center) compared with the control (left) beetle leg. The control beetles were fed with the home-made jelly (no chemical indicator added) for 2 days prior to observation. The error bars represent the standard deviation (S.D.) (N = 5 beetles, n = 30 beetle legs for control, DiBAC_4_(3), Rhodamine B, and Rhodamine 123; N = 9 beetles, n = 30 beetle legs for Fluo-8 and Cell Tracker). Each data set was compared with control leg data set by student’s t-test (Fluo-8, p = 1.42×10^-4^; Rhodamine 123, p = 4.65×10^-5^; DiBAC_4_(3), p = 6.26×10^-9^; Rhodamine B, p = 1.15×10^-9^ and Cell Tracker, p = 7.10×10^-3^). The color scale is given on the bottom left corner of the image. The increase in fluorescence intensity for the chemical indicator-dosed beetle compared with the control beetle indicates that the oral dosing method successfully administers and delivers various chemical indicators in order to label the beetle leg muscle.

The average fluorescence intensity of all the beetle legs (N = 5 beetles, n = 30 beetle legs for control, DiBAC_4_(3), Rhodamine B, and Rhodamine 123; N = 9 beetles, n = 30 beetle legs for Fluo-8 and Cell Tracker) was summarized in Fig. 2. Based on the fluorescence images and the digitized fluorescence intensities that are shown in Fig. 2, we found that the fluorescence intensity increased after oral dosing with all of the tested chemical indicators.

The control beetles were fed with as-is home-made jelly (no chemical indicators added) for 2 days prior to observation. The control beetles’ legs exhibited a certain level of fluorescence intensity (auto-fluorescence), which might be attributed to the organelles that contain fluorescence-emitting molecules, such as NAD(P)H inside the mitochondria [31]. Among the tested chemical indicators, drastic increases in fluorescence intensity were observed for DiBAC_4_(3) and Rhodamine B, while the intensity increases for Rhodamine 123, Fluo-8, and Cell Tracker were relatively small. The differences in fluorescence intensity increase may account for the amount of dye that is retained in the muscle, but we could not confirm this based on our results alone, because different dyes have different properties, such as quantum yields and cellular distribution, as discussed below.

Rhodamine B (QY = 0.65) and Rhodamine 123 (QY = 0.90) have similar high quantum yields in ethanol [32], but Rhodamine 123 showed a small increase in the intensity. This may be attributed to the self-quenching of Rhodamine 123 at higher concentrations, where the quenching mechanism is not yet known. Rhodamine 123 and Rhodamine B are both cationic fluorescent dyes that are localized to mitochondria or acidic organelles such as endosomes and lysosomes, respectively [33–35].

DiBAC_4_(3) is a bis-oxonol, slow-response potential indicator that is lipophilic, negatively charged, and binds to intracellular proteins or membranes of depolarized cells. DiBAC_4_(3) was bright under observation, which was probably due to its properties, of which the quantum yield in aqueous solution is greatly increased once it binds to cytosolic proteins inside of the cell [18, 36–41].

Fluo-8 is a visible light-excitable Ca^2+^ indicator that has a K_d_ for Ca^2+^ of approximately 389 nM with ex/em wavelengths at 490/514 nm, respectively [42, 43]. Fluo-8 has a low quantum yield of 0.16, as measured in 5 mM calcium citrate [44], and thus may appear less bright than the other high quantum yield indicators. Since Fluo-8 binds to intracellular Ca^2+^, monitoring the fluorescence intensity of Fluo-8 would allow us to quantitatively track Ca^2+^ dynamics that are associated, for example, with muscle contraction, which can be induced by electrical stimulation. However, in general, the fluorescence intensity reflects not only the concentration of free Ca^2+^ ([Ca^2+^]) but also the sample’s displacement perpendicular to the focal plane of the microscope.

Cell Tracker was employed as the reference indicator, and Cell Tracker and Fluo-8 were mixed together into a jelly to feed to test beetles. Cell Tracker is a chemical indicator that concentrates in the cytoplasm after penetrating the cell and exhibits fluorescence ex/em wavelengths at 548/576 nm, respectively [18].

In our study, Fluo-8 and Cell Tracker were initially modified with an acetoxymethyl (AM) ester group, which makes the molecules sufficiently hydrophobic so that they diffuse into cells through the plasma membrane. Once these molecules enter into the cell, the intracellular esterase cleaves the AM ester group by hydrolysis, leaving the main group of the indicator molecules [26, 27]. A possible reason for the relatively small increases in the fluorescence intensity for Fluo-8 and Cell Tracker compared with the other chemical indicators could be associated with the AM ester cleavage process as well as the low quantum yield of these dyes. Although ideally the AM ester group is only cleaved inside of the cell by intracellular esterase, the cleavage could also be induced by some extracellular esterase and/or even H_2_O, which might have occurred during the course of delivery of the indicator molecules from the digestive system to the leg muscle cells. If such extracellular cleavage occurs, the indicator molecules (without the AM ester group) cannot penetrate the plasma membrane and thus a smaller quantity of indicator molecules in the cells occurs.

### Ca^2+^ Dynamics Induced by Electrical Stimulation

**Figure 3:**
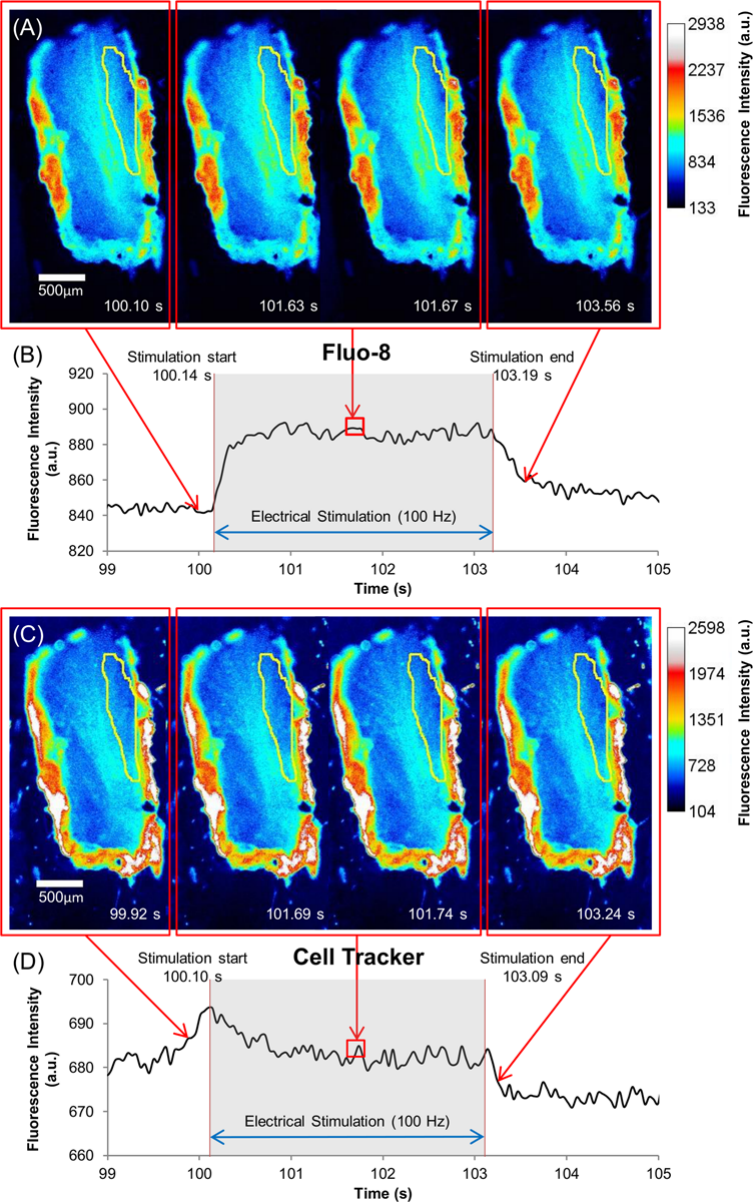
Ca^2+^ dynamics and muscle displacement of the beetle leg muscle under electrical stimulation with multiple pulse trains (100 Hz, 10% duty cycle, 2 V) for 3 s. Pseudocolor time series images of beetle leg muscle dosed with (A) Fluo-8 (60 µM) and (C) Cell Tracker (60 µM) indicators. ROIs are indicated by yellow region (as also shown in S1B Fig.). The color scale is given on the right side of each image (A) or (C) respectively. Fluorescence intensity dynamics of (B) Fluo-8 and (D) Cell Tracker under electrical stimulation, digitized with ImageJ software from the ROI shown in (A) and (C) respectively. The stimulus timing is indicated by grey shading. The Fluo-8 pseudocolor images illustrate the fluorescence intensity dynamics that correspond to the Ca^2+^ dynamics inside the leg muscle: it increased at the start of electrical stimulation, was maintained during the application of the stimulus, and finally slowly decreased after the stimulus stopped. The Cell Tracker pseudocolor images display that the muscle displacement also causes intensity change during electrical stimulation, which slightly affects the Fluo-8 measurement.

The fluorescence intensities of both Fluo-8 and Cell Tracker in the leg of the beetles changed upon electrical stimulation. After the beetle was orally dosed with jelly containing Fluo-8 and Cell Tracker, electrical stimulus of multiple pulse trains (100 Hz, 10% duty cycle, 2 V) was applied to one of the leg muscles of the beetle. Representative pseudocolor fluorescence images and intensity profiles for Fluo-8 and Cell Tracker indicators before, during, and after applying the electrical stimulus are shown in Fig. 3. The Fluo-8 signal in Fig. 3B reveals that there was an increase in [Ca^2+^] owing to the electrical stimulus, and [Ca^2+^] increased and then settled a plateau until the stimulation ended, and then gradually declined afterward. However, muscle contraction displaces the muscle in the direction perpendicular to the focal plane of fluorescence microscope so that the changes in the fluorescence intensity correspond not only to the changes in the [Ca^2+^] but probably also to the displacement of muscle out of the focal plane. Cell Tracker, which is a chemical indicator that is insensitive to Ca^2+^, was employed to estimate the contribution of the muscle contraction (vertical displacement) to the change in fluorescence intensity. The relatively low increase in the fluorescence intensity that was observed with Cell Tracker as a reference (Fig. 3C and 3D) is attributable to the muscle displacement (S2B Fig.).

**Figure 4:**
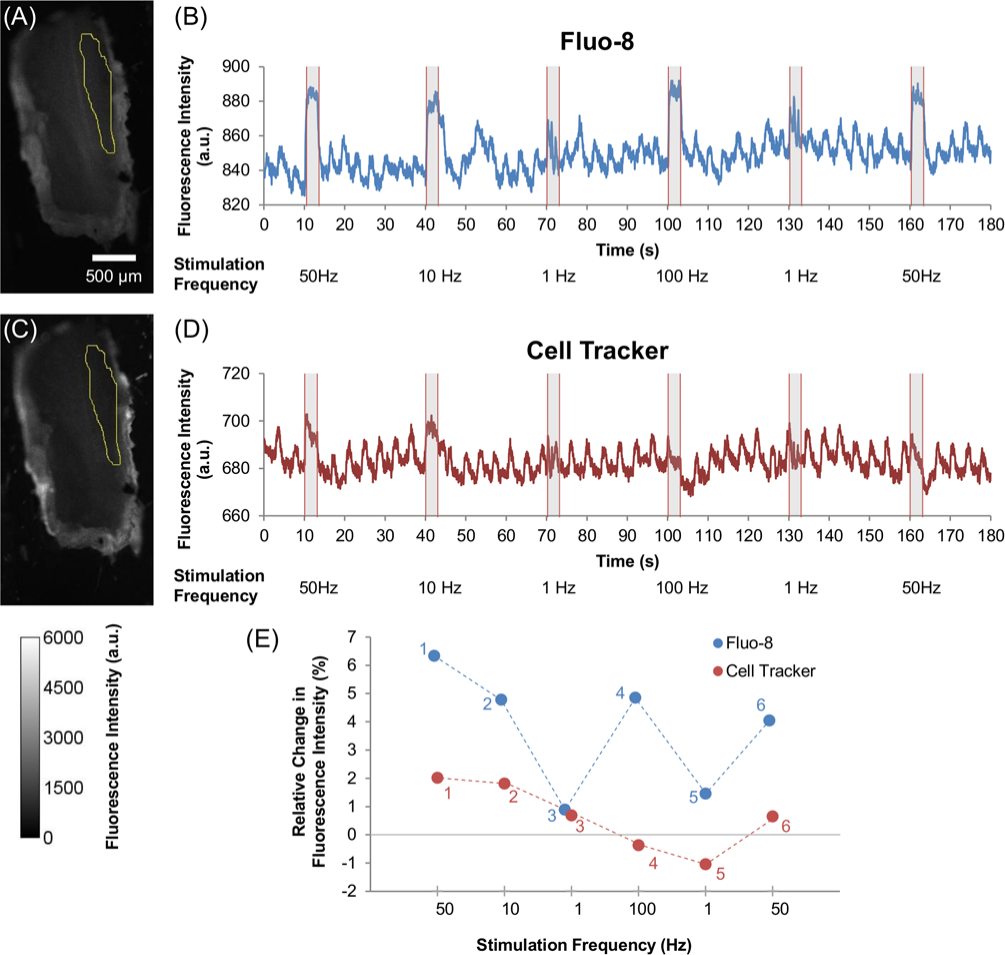
Effect of various electrical stimulation frequencies on Ca^2+^ dynamics and muscle displacement in beetle leg muscle. Images of beetle leg muscle that was dosed with (A) Fluo-8 (60 µM) and (C) Cell Tracker (60 µM), with the yellow selection indicating the ROI that was used for analysis (as also shown in S1B Fig.). The color scale is given at the bottom left corner of the image. Representative time courses showing the fluorescence intensity dynamics of (B) Fluo-8, and (D) Cell Tracker under various electrical stimulations of multiple pulse trains (50 Hz, 10 Hz, 1 Hz, 100 Hz, 1 Hz, and 50 Hz; 10% duty cycle; 2 V) observed from the ROI that are displayed in (A) and (C) respectively. All electrical stimulations were applied for 3 seconds periods with a 27 seconds resting period in between stimulations. The stimulus timing is indicated by grey shading. (E) Relative change in fluorescence intensity ((Δ*F*/*F*_0_)×100%) for Fluo-8 (blue) and Cell Tracker (red) under varying electrical stimulation frequencies (50 Hz, 10 Hz, 1 Hz, 100 Hz, 1 Hz, and 50 Hz; 10% duty cycle; 2 V). The small numbers next to each plot indicate the order of stimulation; i.e., first from 50 Hz followed by varying frequency pulses (10 Hz, 1 Hz, 100 Hz, 1 Hz, and 50 Hz). The Fluo-8 intensity plot shows that Ca^2+^ dynamics are dependent on electrical stimulation frequency, whereas the Cell Tracker plot shows that the muscle displacement contributes a small amount.

**Figure 5:**
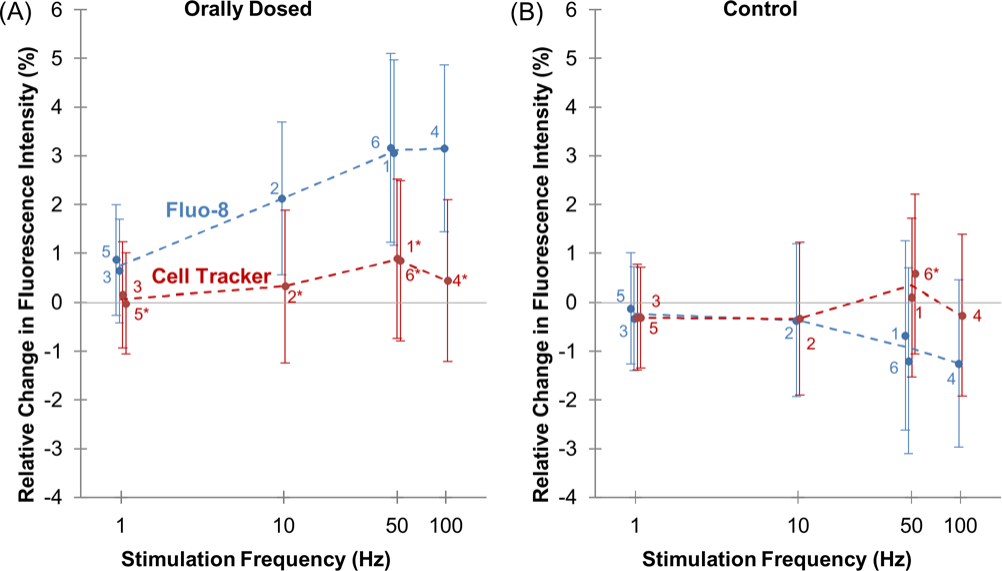
Relationship of Ca^2+^ dynamics with electrical stimulation frequency. Relative changes in fluorescence intensity ((Δ*F*/*F*_0_)×100%) for leg muscle of (A) beetle orally dosed with Fluo-8 (blue) and Cell Tracker (red) and (B) control beetle measured with the filter setting used for Fluo-8 (blue) and Cell Tracker (red) under varying electrical stimulations (1 Hz, 10 Hz, 50 Hz, and 100 Hz; 10% duty cycle; 2 V). Data were analyzed from the ROI adjacent to the stimulated site (S1B Fig.). The error bars represent the S.D. (N = 8 beetles, n = 24 beetle legs for (A); N = 2 beetles, n = 8 beetle legs for (B)). The small numbers next to each plot indicate the order of stimulation. Cell Tracker data set was compared with Fluo-8 data set at each stimulation frequency evaluated by student’s t-test both for dosed beetles in (A) (1st 50 Hz, p = 9.37×10^-4^; 10 Hz, p = 7.45×10^-3^; 1st 1 Hz, p = 2.30×10^-1^; 100 Hz, p = 4.17×10^-4^; 2nd 1 Hz, p = 4.49×10^-2^; and 2nd 50 Hz, p = 8.16×10^-3^) and for control beetles in (B) (1st 50 Hz, p = 4.10×10^-1^; 10 Hz, p = 9.30×10^-1^; 1st 1 Hz, p = 9.29×10^-1^; 100 Hz, p = 5.69×10^-2^; 2nd 1 Hz, p = 6.85×10^-1^; and 2nd 50 Hz, p = 2.67×10^-2^). The significant differences are displayed by an asterisk (p < 0.05). Fluo-8 intensity dynamics show that Ca^2+^ dynamics inside the muscle have a positive correlation with electrical stimulation frequency; i.e., higher stimulation frequency induces larger increase in [Ca^2+^]. On the other hand, Cell Tracker intensity dynamics show that the frequency-dependent intensity change due to muscle displacement is not apparent.

Increase in the fluorescence intensity of Fluo-8 when induced by electrical stimulation was enhanced with increased stimulus frequency, while there was less significant change in that of Cell Tracker, as shown in Figs.4 and 5. The contraction of most muscles is regulated by the rate of physiological neural signal input to the muscle: higher neural input induces larger muscle contraction (tonic contraction) [45, 46]. Similarly, a higher frequency of externally applied electrical stimulus to living muscle induces larger muscle contraction (summation or facilitation) [47–49]. These two types of contraction-enhancement are both due to the elevation of [Ca^2+^] in muscle cells [19, 50]. Thus, we compared the dependency of fluorescence intensity changes (Δ*F*/*F*_0_ defined as below) of Fluo-8 and Cell Tracker on stimulation frequency in order to examine the effect of electrical stimulus on intracellular Ca^2+^ dynamics for each beetle leg. Δ*F* was defined by (*F*_1_ —*F*_0_), where *F*_1_ is the average fluorescence intensity during stimulation and *F*_0_ is that of 10 frames before beginning electrical stimulation, both measured from the ROI adjacent to the stimulated sites (electrode implanted sites) as shown in S1B Fig. To compare Δ*F* of these different chemical indicators, we evaluated the relative changes in Δ*F*, defined as Δ*F*/*F*_0,_ at each stimulation frequency. The Δ*F*/*F*_0_ was then plotted with respect to applied stimulus frequencies in Fig. 4E. Note that the Δ*F*/*F*_0_ of Cell Tracker in some cases displays negative values; for example, during the 100 Hz stimulation and the second 1 Hz stimulation in Fig. 4E, indicating that the muscle displacement in some cases caused decreased intensity.

The Δ*F*/*F*_0_ of Fluo-8 depended on stimulus frequency, while the Δ*F*/*F*_0_ of Cell Tracker was relatively constant throughout the applied stimulus frequencies (Fig. 5A). Besides, such a distinguishable difference in the stimulation-frequency dependency of Δ*F*/*F*_0_ between Fluo-8 and Cell Tracker is not seen for the control beetle (Fig. 5B). Overall, these results indicate that the orally dosed Fluo-8 successfully monitored [Ca^2+^] inside the cell, and the change in the fluorescence intensity of Fluo-8 primarily reflects Ca^2+^ dynamics, whereas the muscle displacement has a rather small contribution to the intensity change.

Periodic fluctuations in fluorescence intensities of both Fluo-8 and Cell Tracker were continuously observed, regardless of applied electrical stimulations (Fig. 4B and 4D). The frequency of the periodic fluctuations was 0.25–0.26 Hz, which is similar to the frequency of tracheal respiration in beetles [51]. The tracheal respiration to supply oxygen caused periodic change in the leg muscle displacement, which resulted in the periodic fluctuations in the fluorescence intensity.

We found that the dependence of the [Ca^2+^] (monitored as Fluo-8 intensity change) on stimulation frequency is reproducible, as shown in Fig. 5. For example, the Δ*F* for the first 50 Hz stimulation (point 1) was reproduced on the last 50 Hz stimulation (point 6), although the muscle experienced different stimulation frequencies in between. This reproducibility indicates that the stimulated muscle was not significantly damaged and it can retain its elasticity and tension, although various stimulation frequencies were applied. Therefore, our stimulation protocol is fairly safe to use repeatedly on *M. torquata* beetles.

The challenge ahead is to substantially increase the fluorescence intensity of Fluo-8 than that of the auto-fluorescence in order to globally measure (visualize) and map out the Ca^2+^ dynamics over the whole muscle. Oral dosing of Fluo-8 in insect leg muscle allowed us to quantitatively estimate the effect of electrical stimulation on Ca^2+^ dynamics (muscle contraction) and observe whether the stimulated muscle has been damaged. However, the fluorescence intensity of Fluo-8 using the current oral dosing method is not sufficient to map out the Ca^2+^ dynamics over the whole muscle, because the auto-fluorescence level is high. We need to modify the oral dosing protocols and modify the molecular structure of the Ca^2+^ indicator such that it resists esterase cleavage, which occurs by hydrolysis, during the transport from the digestive system to the target muscle such that higher quantities of these indicators could diffuse into muscle cells.

## Summary

As a non-invasive and facile staining and labeling technique for insect bioimaging, we proposed and demonstrated oral dosing of chemical indicators by feeding live insects with jelly that contained the chemical indicators of interest. Insect leg muscles were successfully stained by orally dosed chemical indicators including Rhodamine B, Rhodamine 123, DiBAC_4_(3), Fluo-8, and Cell Tracker Orange CMRA. The staining process in the oral dosing method does not require invasive and complicated operations and/or handling, unlike other conventional chemical dosing methods. The Ca^2+^ indicator Fluo-8 was further used to track Ca^2+^ dynamics, and Cell Tracker was used as reference to estimate the contribution of the displacement of electrically stimulated muscle to fluorescence intensity change owing to the displacement of the muscle. We confirmed that the fluorescence intensity increase of the orally dosed Fluo-8 was enhanced by increasing the frequency of electrical stimulus that was applied to the muscle, while that of the orally dosed Cell Tracker was slightly dependent on the stimulation frequency. This result indicates that the orally dosed Fluo-8 successfully monitored Ca^2+^ inside of the muscle cells. This method would allow us to quantitatively investigate the physiological effects of exogenously applied electrical stimulus on living insect muscles. The oral dosing method would work to diffuse foreign materials and globally label molecules and tissues in varieties of insect species. Probably some messenger molecules in neurons in both motor units and sensory units can be labeled simultaneously. In the past years, researchers have been studying the relationship of the motor and sensory units in some insects, for example optic lobes and antennal lobes, in the central nervous system (CNS) of the insects of their interest [52–55]. The development of neuronal Ca^2+^ imaging enabled researchers to further examine the mechanism of neuronal and sensory Ca^2+^ signaling in some animals [56, 57]. Therefore, once the oral dosing method is modified and improved so as to non-invasively and globally but yet selectively stain specific motor and sensory units simultaneously, we can comprehensively investigate varieties of physiological events in CNS: we can study not only specific physiology of individual motor or sensory unit but also the connection and interactions (neural signal propagations) between these units.

## Supporting Information

S1 Fig Regions of interest (ROIs) used to analyze the data. Greyscale images of leg muscle with (A) 2 ROIs and (B) 1 ROI adjacent to the stimulated site. The center part of the leg which is not part of the muscle and the cuticles on the periphery was excluded from analysis. Two yellow dots in (B) indicate the position of the electrodes for stimulation. The stimulation site were either on the right side of the muscle, as depicted in (B) or on the left side of the muscle.
(TIF)

S2 Fig Muscle displacement during electrical stimulation. (A) Yellow rectangle indicates the ROI used to draw a kymograph in (B). (B) Kymograph of muscle taken from the ROI shown in (A) for the time period (as in Fig. 3B) from 99.00 seconds to 105.00 seconds in which electrical stimulus (100 Hz; 10% duty cycle; 2 V) was applied from 100.14 seconds to 103.19 seconds as denoted by the two yellow lines.
(TIF)
