# Supplementary figures and images for: Oral Dosing of Chemical Indicators for In Vivo Monitoring of Ca2+ Dynamics in Insect Muscle
Source: PLoS One. 2015 Jan 15;10(1):e0116655. doi: 10.1371/journal.pone.0116655 (PMC4295878; doi:10.1371/journal.pone.0116655)

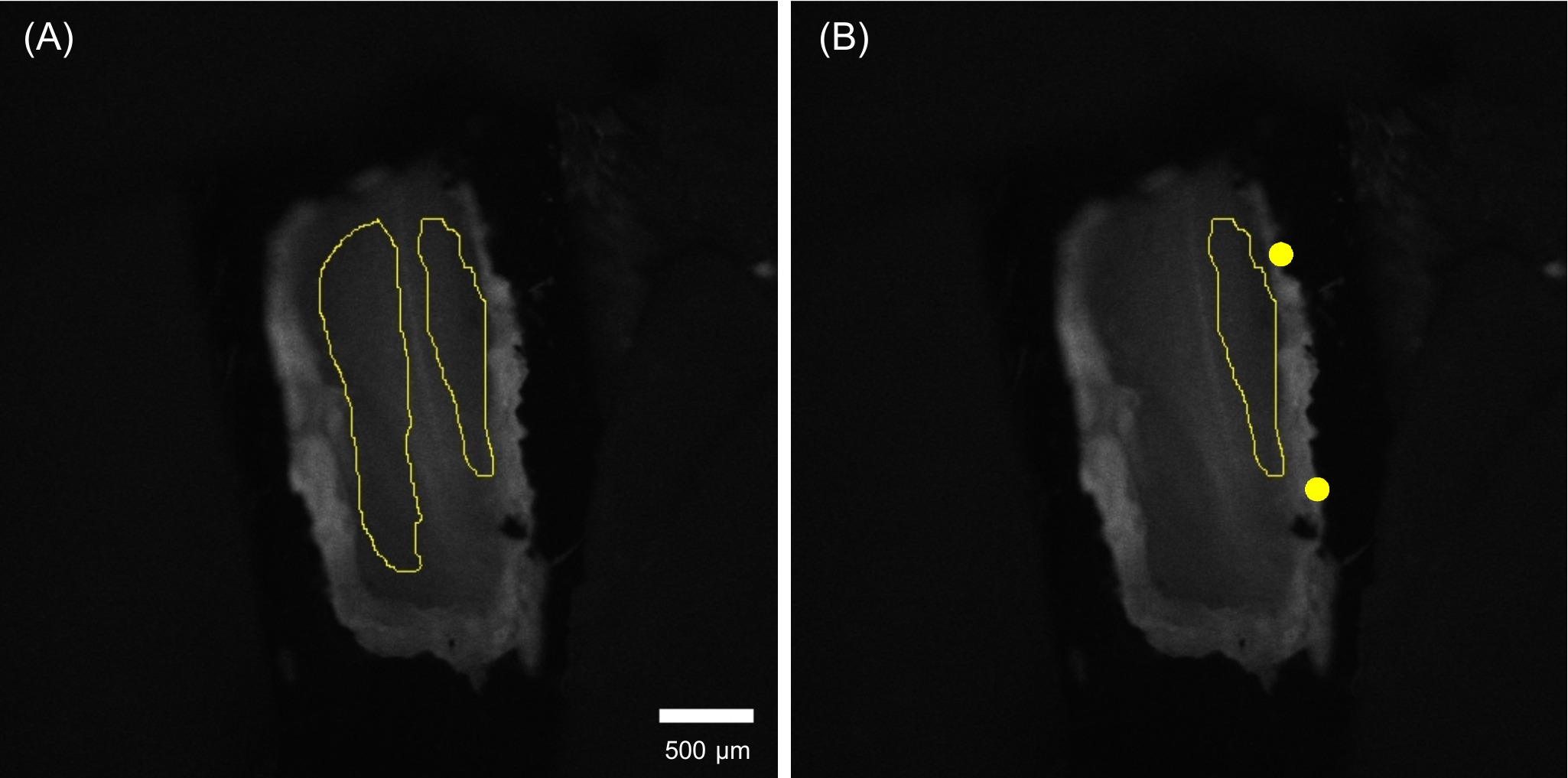

Supplement: S1 Fig — Greyscale images of leg muscle with (A) 2 ROIs and (B) 1 ROI adjacent to the stimulated site. The center part of the leg which is not part of the muscle and the cuticles on the periphery was excluded from analysis. Two yellow dots in (B) indicate the position of the electrodes for stimulation. The stimulation site were either on the right side of the muscle, as depicted in (B) or on the left side of the muscle. (TIF) [file pone.0116655.s001.tif]

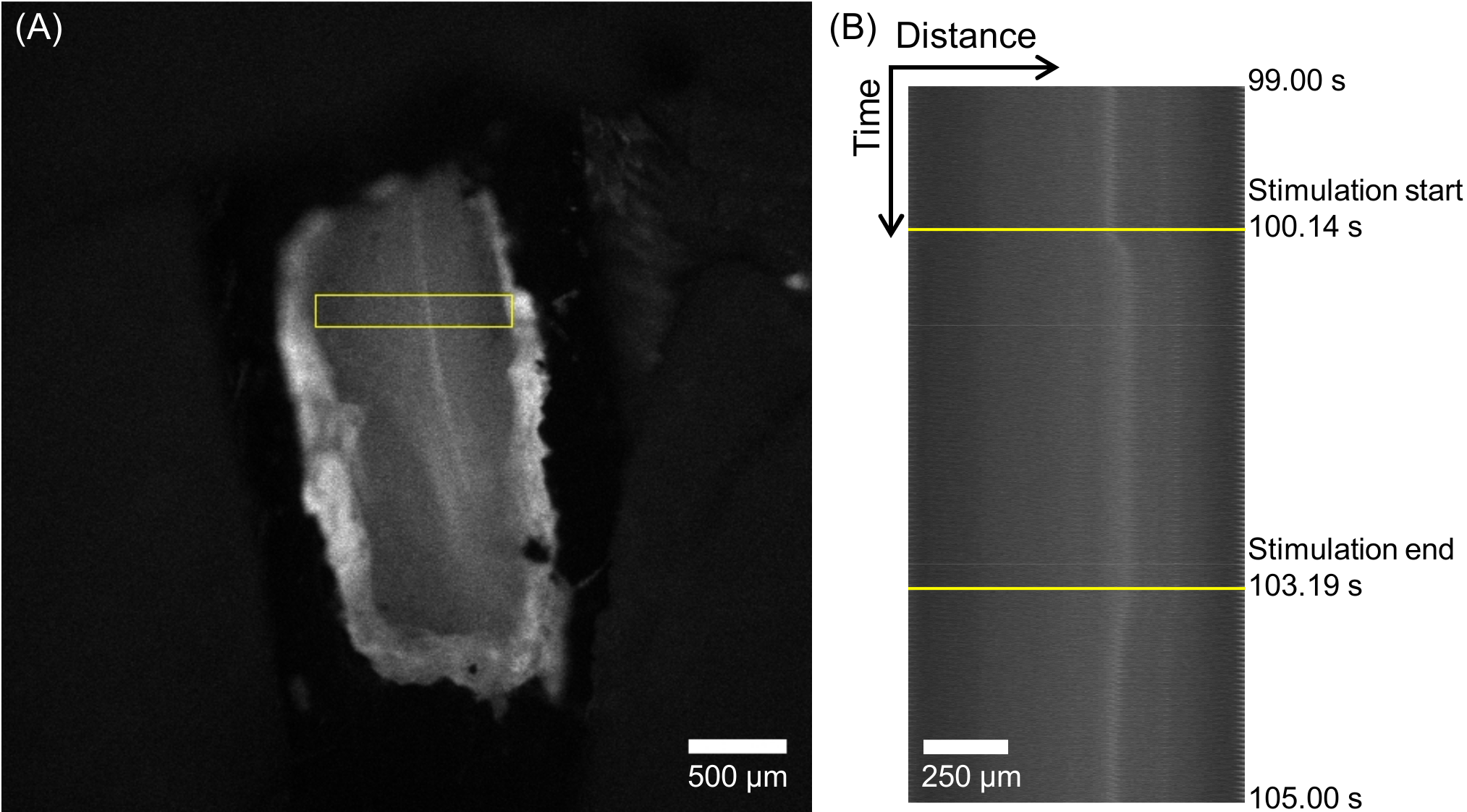

Supplement: S2 Fig — (A) Yellow rectangle indicates the ROI used to draw a kymograph in (B). (B) Kymograph of muscle taken from the ROI shown in (A) for the time period (as in Fig. 3B) from 99.00 seconds to 105.00 seconds in which electrical stimulus (100 Hz; 10% duty cycle; 2 V) was applied from 100.14 seconds to 103.19 seconds as denoted by the two yellow lines. (TIF) [file pone.0116655.s002.tif]
